# Prevention of posterior capsule opacification through intracapsular hydrogen peroxide or distilled water treatment in human donor tissue

**DOI:** 10.1038/s41598-018-31178-y

**Published:** 2018-08-24

**Authors:** Justin Christopher D’Antin, Rafael I. Barraquer, Francisco Tresserra, Ralph Michael

**Affiliations:** 1 Institut Universitari Barraquer, Universitat Autònoma de Barcelona, Barcelona, Spain; 2 Centro de Oftalmología Barraquer, Barcelona, Spain; 3 Universitat Internacional de Catalunya, Barcelona, Spain; 4 Department of Pathology, Institut Universitari Dexeus, Barcelona, Spain

## Abstract

In order to determine whether posterior capsule opacification after cataract surgery, could be delayed or inhibited through the application of hydrogen peroxide (H_2_O_2_) or distilled water (H_2_Od),we extracted lens capsules from 25 human donor eye globes. Samples were treated for 5 min with either 30 mM H_2_O_2_ or H_2_Od or used as controls, and cultured for one month, during which dark field and tilt illumination photos were taken. These were used to observe and quantify, time until cellular growth and confluence on the posterior capsule. After culture, histological sections were stained for H&E, α-SMA, Ki-67 and vimentin and evaluated. We prevented cellular growth in 50% of H_2_Od and 58% H_2_O_2_ of treated samples. The overall prevention of cell growth compared to cultured controls was significant for both treatments while there was no significant difference between them. In the cases where cellular growth was not prevented, both treatments significantly delay cellular growth. Until day 28 none of the treated samples of either type that had shown growth reached total confluence. All cultured controls reached total confluence before treated samples (median = day 11.5). Also, histologically, there was a clear morphological difference between cultured controls and treated samples.

## Introduction

The development of posterior capsule opacification (PCO) is due to a combination of the processes of proliferation, migration, and transdifferentiation of residual lens epithelial cells (LECs) on the lens capsule, after cataract surgery, resulting in light scatter on the visual axis^1^.

In the past decades, many approaches for PCO prevention have been studied, including adjustments of surgical techniques, intraocular lens materials and design, pharmacological treatments, and prevention by interfering with biological processes (such as TGF-β signaling, proliferation, migration or membrane stability) in LECs^2–8^. So far the most effective method seems to be the implantation of an intraocular lens with sharp edged optics to mechanically prevent PCO formation^8^.

The use of mechanical methods for the prevention of PCO might not be the best option since it limits the design of Intra ocular lenses (IOLs) and does not prevent PCO in all cases. If PCO could be prevented by chemical or pharmacological treatments, new IOLs or lens refilling techniques could be used or designed that would allow visual accommodation, eliminating the need for corrective multifocal glasses or lenses^9,10^.

Different substances have been studied for the prevention of PCO, such as 5-Fluorouracil^11,12^, Mitomycin-C^13^, Pirfenidone^14^, Rapamycin^15^, Ricin^16^, etc., all with varying degrees of success in the prevention or postponement of PCO. Duncan G. and Wormstone I.M. studied thapsigargin^11,17^ which seems to be the most effective substance to date, leading to total lens cell destruction; however thapsigargin is cytotoxic and can damage the cornea^18^.

To safely and effectively search for preventative substances, we decided to use tissue cultures. Although animal models and LEC cell line cultures are useful, animal models are more difficult to use and LEC cell line cultures are missing the anatomical conditions of the human eye. In this respect, tissue cultures of human capsular bags have advantages, mimicking surgical conditions *in vivo* and allowing growth of LECs on their preferred natural substrate, the lens capsule. The normal anatomical relationships of the capsule and LECs need to be preserved as closely as possible, if the findings of tissue culture models are to be applicable clinically^19^.

We decided to test two basic substances for PCO prevention, distilled water (H_2_Od) and hydrogen peroxide (H_2_O_2_). H_2_Od since it has been tested many times before^11,13,17,20–22^ and gives us a point of reference with other similar studies, although its effectiveness has been inconsistent. H_2_O_2_ was selected due to its known presence in the lens^23^ and its dichotomous role in mammalian cells, being harmful or beneficial depending on its location and concentration. For example, rabbit lens epithelial cells exposed to different concentrations of H_2_O_2_, result in cell death or proliferation depending on concentration^24^. Low levels of H_2_O_2_ have also been shown to promote faster wound healing in cornea both *in vitro* and *in vivo*^25^.

## Material and Methods

The research adhered to the tenets of the Declaration of Helsinki on research involving human subjects. The experimental protocol was approved by the Ethical Committee for Clinical Research of the Centro de Oftalmología Barraquer (Study code: CultivoOCP2015-2018).

Donor eye globes were obtained from the “Banc d´Ulls per a Tractaments de Ceguesa” (BUTC). Written informed consent for the removal and use of the eye globes for diagnostic and research purposes was obtained from patients and/or relatives. In the cases that, after BUTC analysis, the eye (cornea) was classified as non-suitable for transplantation, and as long as the reason did not affect our tissue culture, we proceeded with its use.

A total of 25 donor globes were used, consisting of 3 uncultured & 7 cultured controls, 7 treated with distilled water (H_2_Od) and 8 treated with hydrogen peroxide (H_2_O_2_).

### Sample preparation

The capsular bag–ciliary body complex was dissected in our laboratory under standard biosecurity conditions based on the model proposed by Cleary G, *et al*.^19^. The corneoscleral disk was removed from the globe with a circular trephine, and then the iris–ciliary body–lens complex was dissected from the globe in a single piece.

Specimens were transferred to sterile Petri dishes and placed posterior side up. Any residual adherent vitreous was removed from the posterior surface of the lens, being careful not to breach the posterior capsule. In some cases the vitreous membrane (hyaloid) was torn, leaving remnants on the posterior capsule.

**Figure 1:**
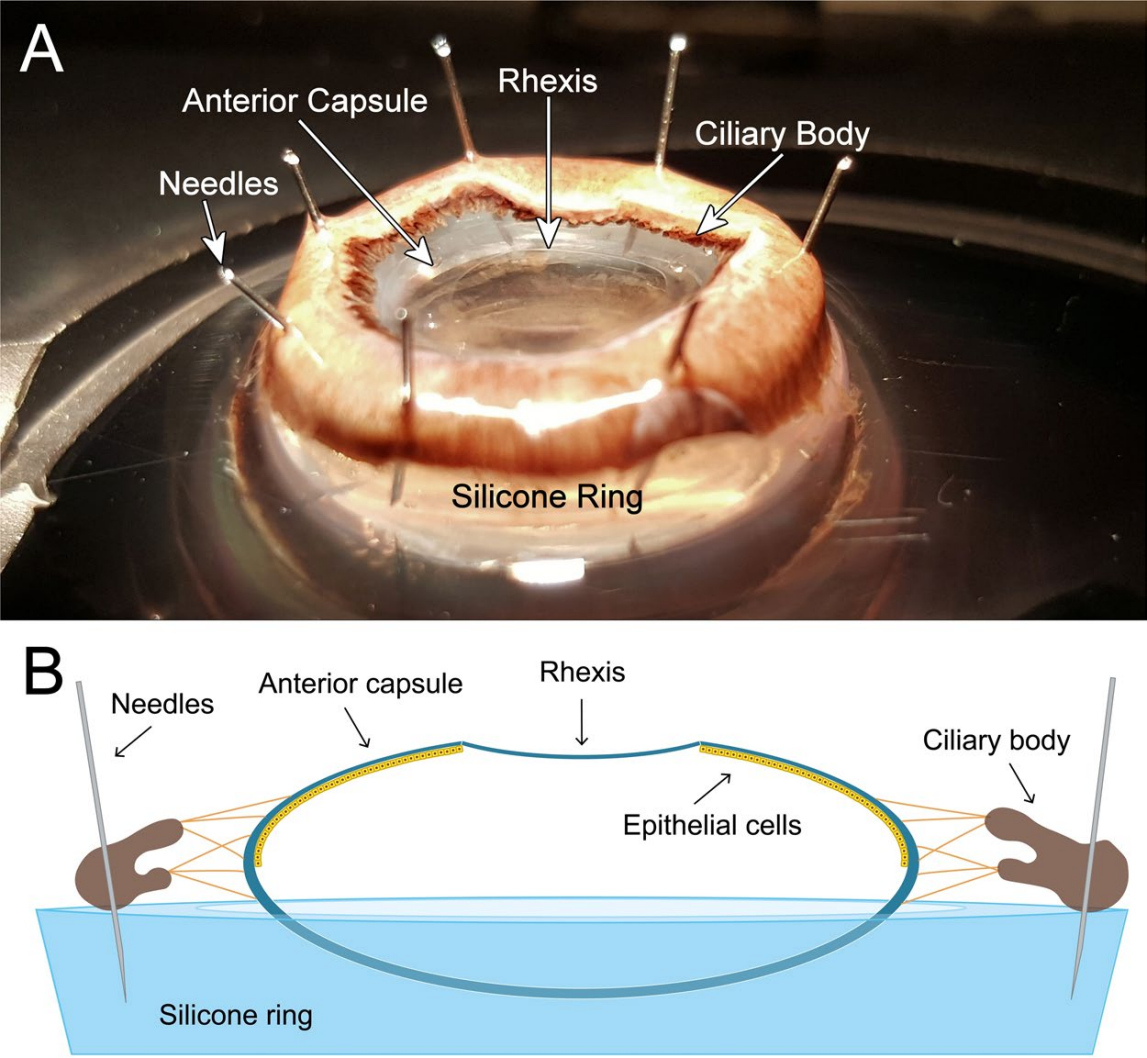
(**A**) Image of a prepared capsule-ciliary body complex fixed to a silicone ring before treatment or being placement in culture media. (**B**) A schematic diagram illustrating a prepared sample and the distribution of lens epithelial cells.

For each sample a silicone ring mount was placed in a deep Petri dish containing balanced salt solution (BSS). The specimen were placed on the silicone ring, anterior surface facing upward, and attached by passing 8 G30 needles through the ciliary body. Afterwards the iris was removed with forceps (Fig. 1).

Finally the anterior capsule was breached with capsulorhexis forceps and a 6 mm continuous circular capsulorhexis was performed. Hydrodissection and hydroexpression were employed and residual lens fibers removed manually with a non-toothed forceps or aspirated with a Simcoe cannula. No IOLs were implanted in the capsule to prevent variations in the results due to the mechanical prevention of cell progression. At this point, the 3 uncultured controls were fixed in 4% paraformaldehyde, for histological analysis. We did this in order to observe the starting point of our cultures and to assure that we started from a LECs monolayer. (Supplementary Fig. S1 and Table S1).

### Sample Treatment

**Figure 2:**
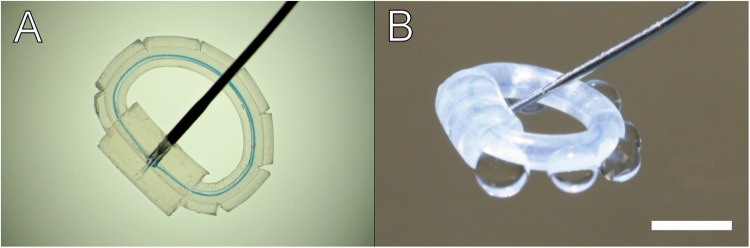
(**A**) The silicone irrigation ring we designed to treat our samples. (**B**) Visualization of treatment solution distribution from ring. Scale bar = 2 mm.

Treatments were performed using an irrigation device designed by our group (Fig. 2). This device is not intended for clinical use. A sealed capsule irrigation device such as the PerfectCapsule™ (Milvella Pty. Ltd.) would have been preferable, but it is no longer on the market. Such a device would avoid contact of the treatment substance with the surrounding tissue^21^. Our device consists of a 16 mm silicone tube (1 mm outer diameter), tied in to a ring with an inner suture and covered with a second 3 mm silicone tube (2 mm outer diameter) where the 2 ends of the first tube meet. Eight evenly spaced cuts are made around the tube to permit uniform liquid distribution. Afterwards it is attached to a syringe, with a blunt needle, containing the treatment substance. Finally the device is introduced in to the capsular bag through the rhexis. This device assures a uniform distribution of the treatment substance within the capsular bag.

Treatments of samples were performed in the absence of BSS to avoid dilution. Samples were treated with 30 mM H_2_O_2_ or H_2_Od during 5 min, after which, samples were thoroughly washed with BSS to remove the treatment. Cultured control samples were only irrigated with BSS.

### Tissue culture

All samples once prepared and treated were transferred to a biosafety cabinet (Bio-II-A, Telstar_®_) and washed 3 times with a BSS and antibiotic/antimicotic solution for 3 minutes. After the third wash, samples were placed in RPMI-1640 culture medium (R8758 Sigma-Aldrich) supplemented with 5% fetal bovine serum (F7524 Sigma-Aldrich) and 1% antibiotic-antimycotic (A5955 Sigma-Aldrich).

The culture medium was exchanged every 2 to 3 days, and LEC growth and migration across the posterior capsule was monitored and documented using an inverted phase contrast microscope (Axiovert 100, Zeiss). Samples were to be cultured for one month.

### Culture monitoring

**Figure 3:**
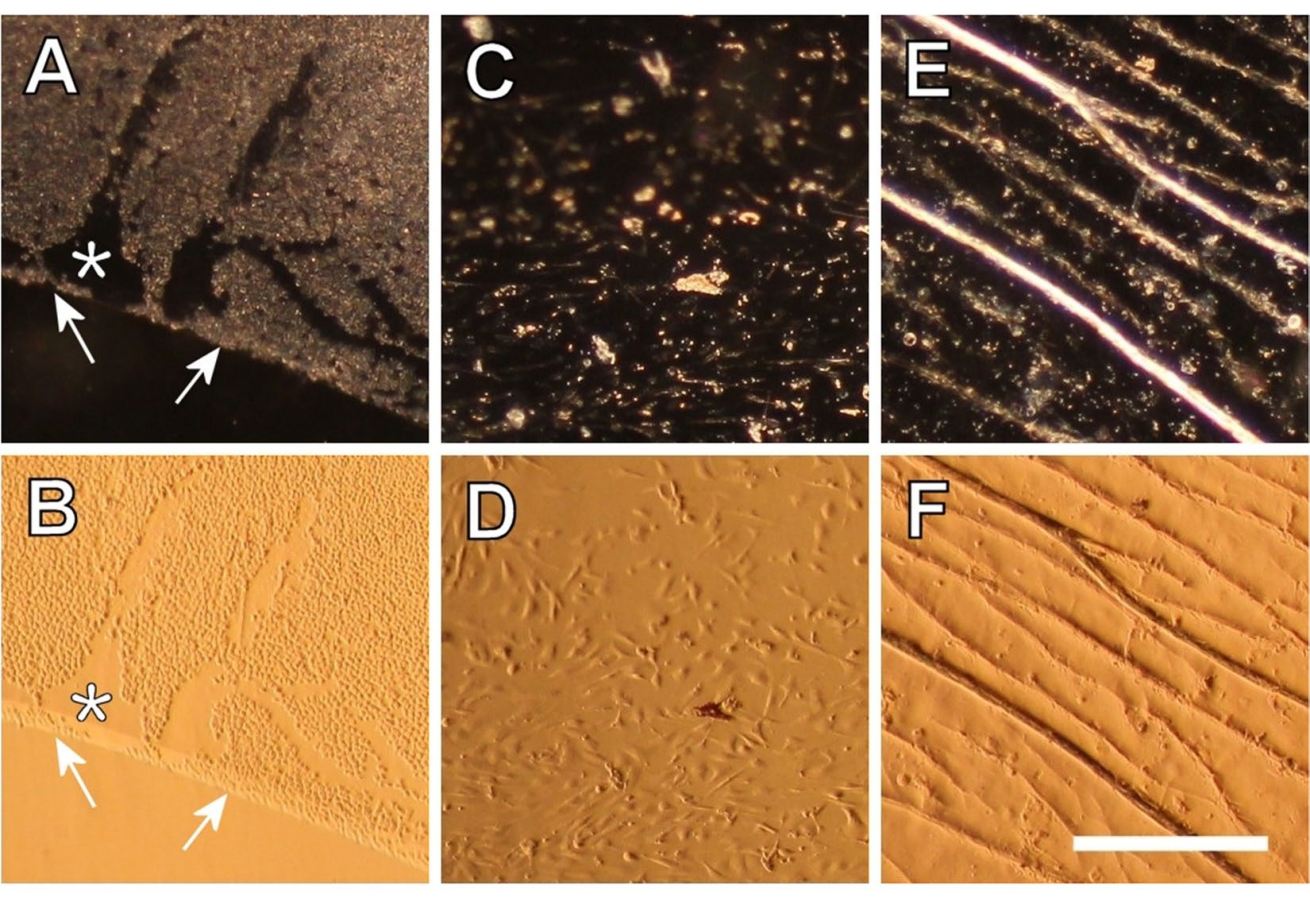
(**A**,**C** & **E**) Dark field and (**B**,**D** & **F**) tilt illumination images of cultured control samples, showing different types and stages of PCO. (**A**,**B**) Initial organized distribution of LECs on the anterior capsule and clear posterior capsule. Arrows indicate rhexis border. Asterisk shows area where LECs where accidentally scraped off during hydrodissection. (**C**,**D**) Migrated cells on the posterior capsule of a sample after 36 days in culture. (**E**,**F**) Wrinkles produced by cells on the posterior capsule of a sample after 33 days in culture. Scale bar = 400 µm.

Photographs were taken with the microscope, using phase contrast (Ph2) illumination with a normal 2.5x Plan-objective, giving dark field images (Fig. 3A,C and E). This allowed for the evaluation of relative transparency of the capsule and migration patterns of LEC.

**Figure 4:**
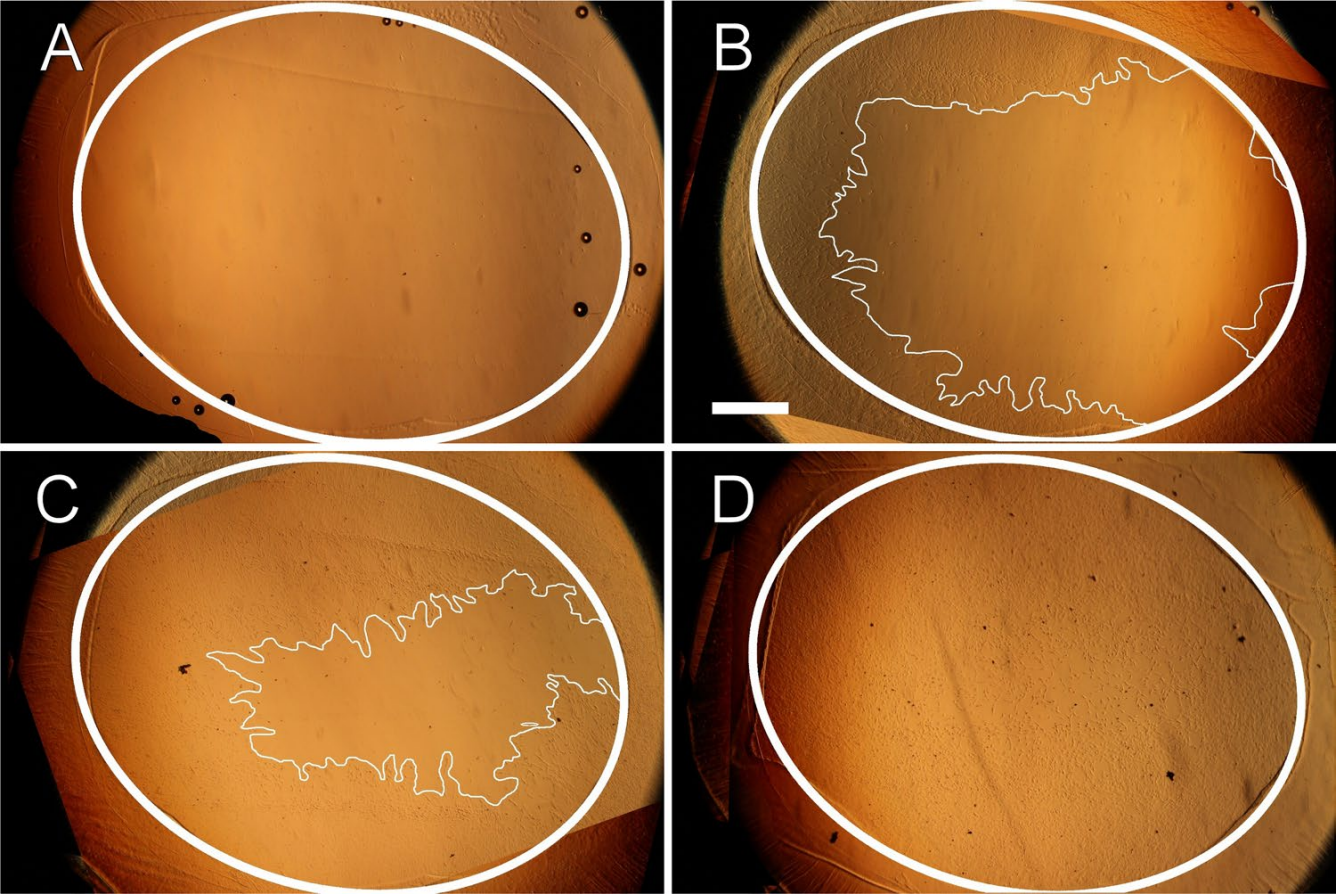
Tilt illumination images of cellular growth progression on the posterior capsule of cultured control sample C031 on (**A**) day 1, (**B**) day 8, (**C**) day 10 and (**D**) day 17 of culture. The thicker white line indicates the rhexis border and the thinner white line indicates the border of progression used to calculate total area coverage on the posterior capsule and speed of growth. Scale bar = 1 mm.

Photographs of the same regions were also taken with the phase contrast illumination ring slightly misaligned (Technique previously described by “Eldred *et al*.” and named modified dark field)^26^; we termed these “tilt illumination images” (Fig. 3B,D and F). This technique gives better images of the individual cells, facilitating the visualization of cellular migration and morphology on the posterior capsule (Fig. 4).

With these imaging techniques, we could distinguish different aspects of PCO, such as cellular distortions; small clusters of disorganized lens cells on the posterior capsule that scattered light in dark field images (Fig. 3C,D) or wrinkles; folds on the posterior capsule caused by lens cell migration and myofibroblastic differentiation resulting in visual distortions^3^ (Fig. 3E,F).

**Figure 5:**
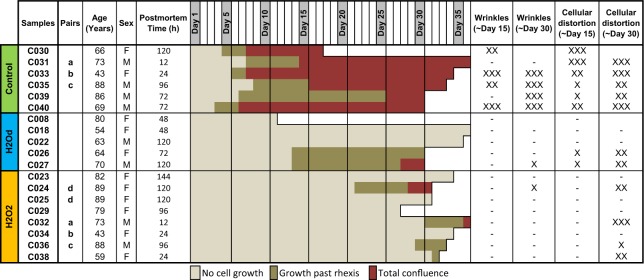
Relevant Information associated with samples used in this study and summarized results of culture. Samples with matching letters are pairs. F = Female, M = Male. The amount of wrinkles and cellular distortion on the posterior capsule were based on tilt illumination and dark field images, on a scale from 0 (−) to 3 (XXX).

We subjectively quantified the degrees of these distortions in order to asses PCO severity. Four degrees of wrinkles and cellular distortions were defined: transparent (−), low (X), medium (XX) and high (XXX) degree of either wrinkles or cellular distortions (Fig. 5). We based this scale on the lowest and highest degrees of distortions observed in our samples.

We defined cellular growth as the presence of cells on the posterior capsule within the visual limits of the anterior rhexis, termed rhexis area. Once the rhexis area was totally covered in cells it was considered that total confluence was reached.

The area between the rhexis and the border of cell progression was considered the area of cell coverage. This area was calculated by outlining the rhexis and the border of migrating cells on the posterior capsule in all sample images as seen in Fig. 4. These images were then processed using ImageJ^27^, giving the area in pixels which we passed to mm^2^.

Confluence speed was calculated as increase of area of cell coverage over the respective time interval and expressed in mm^2^/day.

### Histological staining

Immediately after samples were removed from culture, they were placed in 4% paraformaldehyde for fixation for at least one week and then embedded in paraffin. Once all samples were in paraffin, they were sectioned along the sagittal plane and stained on the same day. Tissue were stained with hematoxylin and eosin for morphological analysis, α-smooth muscle actin α-SMA (clone 14A, Cell Marque) for myofibroblast formation and intercapsular adhesion^28–30^, Ki-67 (clone 30–9, Ventana) for proliferation^31^ and vimentin (clone V9, Ventana) a marker of both undifferentiated lens epithelium as well as differentiating fiber cells^32^. All immunostainings were done with the BenchMark® ULTRA device (Ventana Medical Systems, Inc.). We also included negative immunostain controls to support the validity of our stains and identify possible experimental artefacts or background noise. These controls were processed in the same manner with the automated staining device but without the primary antibodies. Capsular adhesion, cellular morphology and the degree of staining of α-SMA, Ki-67 and vimentin were studied at the equatorial region of the capsule and at the center of the posterior capsule.

### Statistics

For the statistical analysis of treatment efficacy, the Kaplan-Meier method with Log rank test was used. We assigned cellular growth or total confluence as end point events. A p-value of ≤0.05 was considered as significant.

## Results

**Figure 6:**
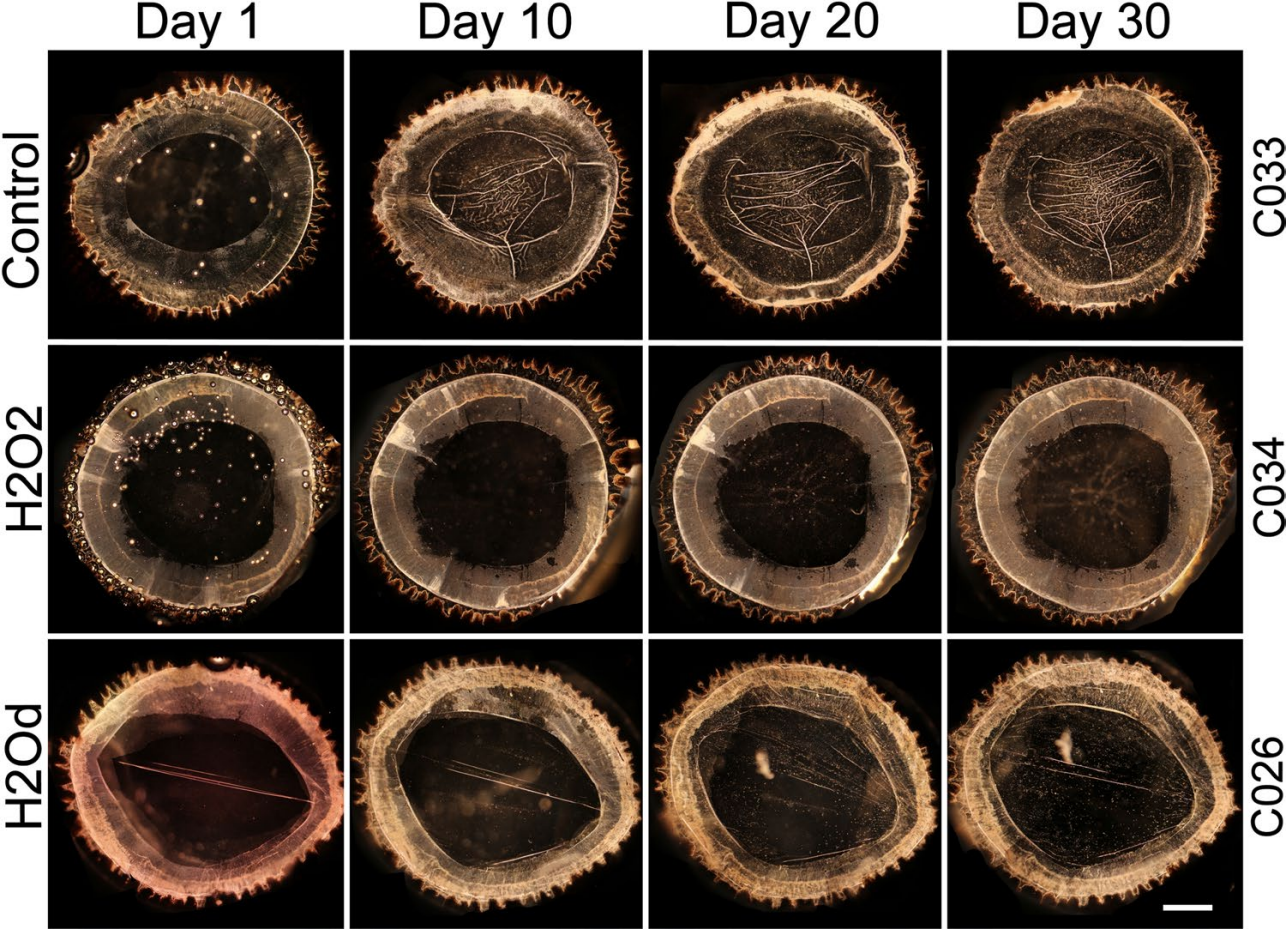
Dark field images of three samples on days 1, 10, 20 and 30 of culture. Cells clusters at the equator and wrinkles can be seen on the cultured control sample. Transparent posterior capsule can be seen throughout the whole H2O2 sample. Scattered cellular growth can be seen on the H2Od sample day 30. The wrinkles that can be seen in H2Od sample is due to the uneven tension of the support needles, not cell migration. Scale bar = 2 mm.

Tissue sample information and a general overview can be seen in Fig. 5. Samples were cultured for an average of 33 days, excluding samples C030, C008 and C029. These didn’t reach the planned 30 days of culture but still gave us valuable data. All cultured control samples showed signs of cellular growth within the first week of cell culture. Some H_2_Od samples started showing signs 2 weeks in and H_2_O_2_ samples only started showing some signs of cellular growth 3 to 4 weeks in. The degree of wrinkles and cellular distortions was much higher and more severe in cultured controls than in treated samples (Fig. 5). Visual examples of these degrees can be seen in Fig. 6, at day 30: C034 is transparent (−) and shows no wrinkles or cellular distortions, C026 shows no wrinkles (−) but a medium degree (XX) of cellular distortions and C033 shows a high degree (XXX) of both wrinkles and cellular distortions. One cultured control and two H_2_Od samples were contaminated before day 5 and no useful results could be obtained so they were discarded.

Most cultured control samples showed medium amounts of wrinkles on the posterior capsule by day 10 while all showed signs of cellular distortions. As culture time progressed the amount of wrinkles and cellular distortions increased. As cells start to migrate; they first concentrate on the equator of the capsule (Fig. 6). Sometimes these cells agglomerate in to clusters of cells that seem to be the first stages of Soemmering ring like formations.

Only one sample from each of the treatment types showed signs of wrinkles and these in low amounts. Varying amounts of cellular distortions were visible in all treated samples that showed signs of cellular growth. An example of cellular distortion can be seen in Fig. 6, H_2_Od days 20 and 30. The H_2_O_2_ treatment kept the posterior capsule transparent in half of the samples, including the sample shown in Fig. 6.

All cultured control samples showed signs of cellular growth on the posterior capsule on average by day 6, 2 of 5 H_2_Od samples showed growth on day 14 and 4 of 8 H_2_O_2_ samples showed growth on average on day 29 (Range: day 22–32) (Fig. 5).

Cellular growth was prevented in all treated samples on day 10. On day 20, prevention was successful in half of H_2_Od and all H_2_O_2_ samples. On day 30, prevention was still successful in half of H_2_Od samples while it had failed in 2 H_2_O_2_ samples. The overall prevention of cellular growth compared to cultured controls was significant for both treatments according to Kaplan Meier statistics (H_2_Od p = 0.001 and H_2_O_2_ p ≤ 0.001) while there was no significant difference between treatments.

Total confluence occurred in all cultured controls on median by day 8 (Range: day 7 to 26), one H_2_Od sample reached total confluence on day 28 and 2 H_2_O_2_ samples reached total confluence on days 29 and 36 (Fig. 5). The overall prevention of confluence compared to cultured controls was significant for both treatments according to Kaplan Meier statistics (H2Od p = 0.002 and H_2_O_2_ p ≤ 0.001) while there was no significant difference between treatments.

The average rhexis area was 31.4 mm^2^ (Range: 23.0 to 35.5 mm^2^), due to this variation, in order to compare confluence progression, we interpreted the area of cell coverage as a percentage of rhexis area. A graph expressing the increase in rhexis area coverage over time in all samples can be seen in supplementary Figure S2. The average speed at which samples reached total confluence was similar among cultured controls (3.2 mm^2^/day ± 0.9) and H_2_O_2_ treated samples (3.0 mm^2^/day ± 2.2) (95% confidence interval). Confluence speed in H_2_Od treated samples was slower (2.0 mm^2^/day ± 0.3).

**Figure 7:**
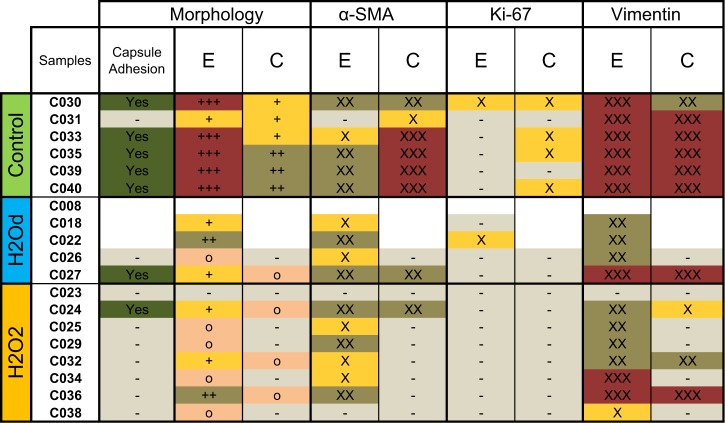
Overview containing all relevant information obtained from histological stains. Intercapsular adhesion between anterior and posterior capsule was indicated as present (yes) or not (−). Observations were made at the equator (E) and the central posterior capsule (C). Cellular Morphology was classified as “clusters or stacked layers of apparently normal cells” (+++); “dispersed, but apparently intact cells with nuclei and cytoplasm” (++); “nuclei without visible cytoplasm” (+); “only residual cell fragments” (o) or “absence of cells and fragments” (−). The fractions of cell content stained with α-SMA, Ki-67 or Vimentin were classified as “all or large majority” (xxx), “about half” (xx); “few” (x) and “none” (−). Samples C008, C018 and C022 are lacking the majority of their histological results due to an error during their sectioning (positions blank).

**Figure 8:**
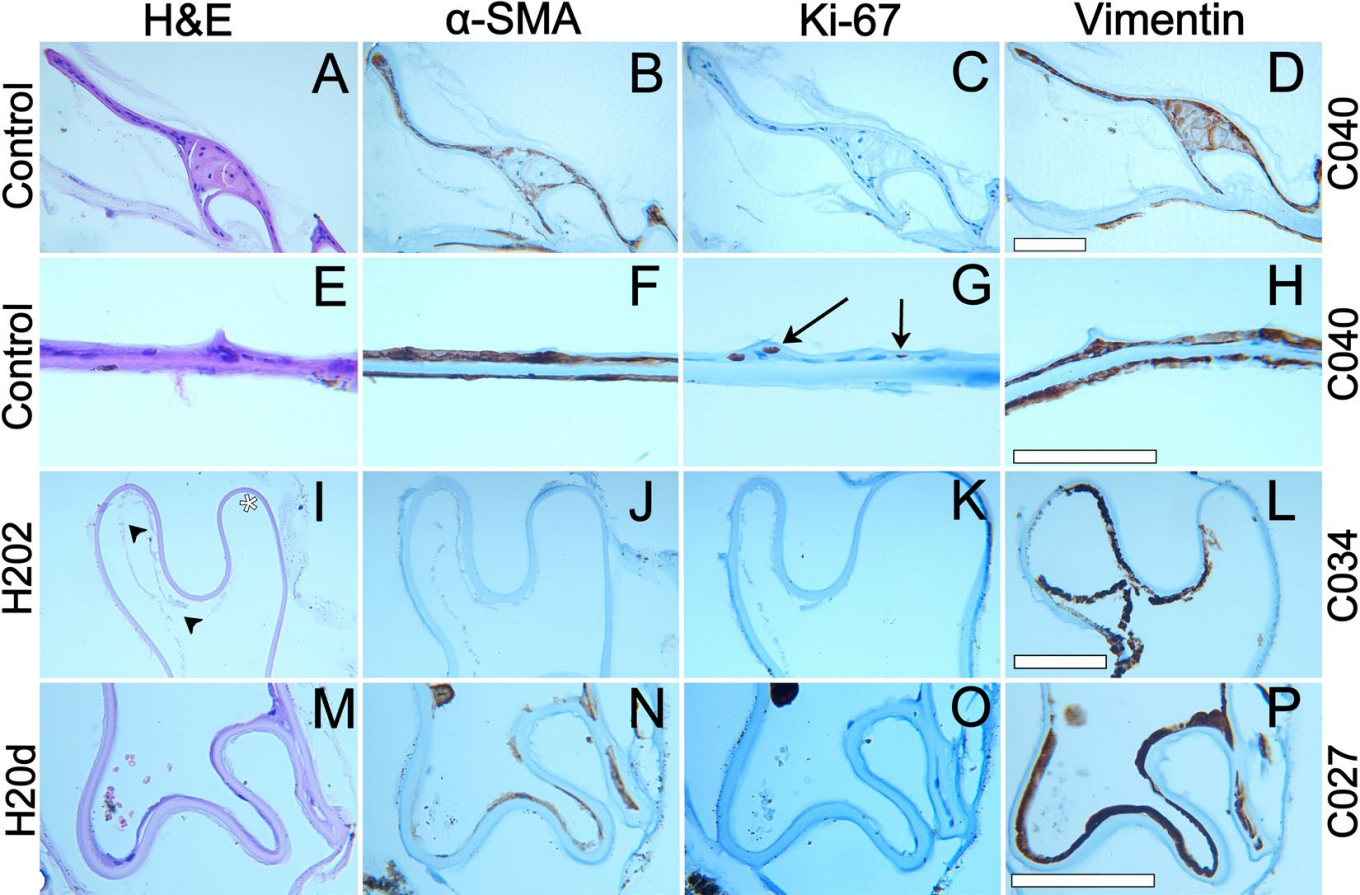
Microscopic images of the histological sections of some of our samples, showing staining for hematoxylin and eosin (**H**,**E**), alpha smooth muscle actin (α-SMA), ki-67 and Vimentin. Images (**A**–**D**) show the equator of cultured control sample C040. Images (**E**–**H**) show the central posterior capsule of sample C040. Images (**I**–**L**) show the equator of H2O2 sample C034. Images (**M**–**P**) show the equator of H2Od sample C027. Arrows highlight Ki-67 staining in image (**G**). Arrowheads show residual cell fragments (**I**). Asterisks highlight an area free of cells (**I**). Scale bars = 100 µm.

All cultured controls (except C031), H2Od sample C027 and H2O2 sample C024 presented intercapsular adhesion. The cellular morphology of uncultured control samples consisted of a single continuous LEC monolayer, while cultured control samples along the equator consisted of stacked layers of cells in all samples except C031 which only presented a monolayer. Cells were also present on the central posterior capsule of all cultured controls. Cellular morphology in treated samples was similar for both treatments; showing some dispersed cells and nuclei at the equator but manly residual cell fragments. These fragments were also present on the central posterior capsule of 4 of the 6 treated samples that had shown cell growth (Figs 7 and 8).

Histologically all cultured controls stained for α-SMA at the equator and more intensely on the center of the posterior capsule, except one (C031). This cultured control sample was the only one that does not show capsular adhesion or wrinkling (Figs 5 and 7). Uncultured controls were negative for α-SMA (Fig. S1). Most treated samples showed α-SMA staining but to a lesser degree and even on cellular fragments (Fig. 7). Ki-67 was present on the posterior capsule of 4 of the 6 cultured controls, showing that cells still proliferate after confluence on the posterior capsule. One uncultured control showed Ki-67 staining at the equator (Fig. S1). Only one treated sample (C022) showed signs of Ki-67, indicating that proliferation had been delayed but not totally prevented and given time this sample would probably have developed PCO. Vimentin was present on all samples with either cells or cell fragments (Figs 7, 8 and S1). All negative immunostain controls confirmed the absence of background noise (Fig. S3).

## Discussion

Overall both distilled water and hydrogen peroxide had a clear effect on PCO, delaying it or in about half of the samples preventing it for 30 days or more.

The variations between samples due to sample preparation, such as rhexis size and removal of lens fibers from within the lens capsule, could have had effects on the initial number or concentration of cells. We tried to mitigate these variables through the random distribution of samples in the different groups, and the use of paired samples in different groups. The three uncultured control samples also only showed a monolayer of LECs without lens fibers, indicating that lens fiber cell removal was complete and consistent. Some contralateral samples were used as cultured controls and their pairs were treated with H_2_O_2_. The significant difference between their cellular growth times supports our hypothesis that H_2_O_2_ can help prevent PCO. Donor age, sex or the postmortem time of the samples had no apparent effects on the results.

A previous study^24^ used rabbit lens epithelial cell lines to evaluate the effects of hydrogen peroxide exposure. Showing that high levels (1 mM) of hydrogen peroxide killed cells and sub lethal levels (100 μM) suppressed their proliferation. While unexpectedly from 1 nM to 1 μM of hydrogen peroxide, there was a dose dependent increase in the cell numbers. Our 30 mM H_2_O_2_ treatment concentration was based on this previous study^24^, however due to the large difference in treatment time (5 min instead of 48 h) our final concentration of 30 mM was an approximately proportional increase to the 100 µM used to suppress LEC line cultures. Furthermore, a study by Tholozan F. *et al*.^33^ indicated that the lens capsule is a source of essential survival factors for LECs, making them more resistant to substance induced apoptosis then LECs cultured in the absence of the capsule, such as in the rabbit study^24^, in which case, a higher concentration of H2O2 is needed in order to obtain the same results.

H_2_O_2_ has the benefit of not only being an endogenously produced substance^23^; it has also been shown to have beneficial effects on the cornea in a similar study^25^, reducing the risks of post-operative corneal complications. Low levels (10–50 µM) of H_2_O_2_ in culture for 48 hours, stimulated adhesion, migration, and faster wound healing on rabbit and pig corneal epithelial cells both *in vitro* and *in vivo*.

However, corneal endothelium is more delicate than corneal epithelium and lacks regenerative capabilities. There is a normal physiological concentration of around 0.5 mM H2O2 in the aqueous humor^34^. Fluctuations of up to 3 fold concentration of H2O2 can be regulated by endogenous catalase^35^. At higher concentrations, the corneal endothelium is in danger of being damaged. This is why our treatment is meant to be applied with a sealed capsule irrigation device, thus avoiding contact of the treatment with the surrounding tissues.

These studies together with our results seem to indicate that it is possible that an ideal concentration of H_2_O_2_ could be found, that suppresses proliferation of LECs while not putting corneal cells in danger or even helping speed up corneal wound healing due to cataract surgery.

Our results can be considered representative of naturally occurring PCO, since the cellular transformations expected in cultured control samples due to epithelial–mesenchymal transition are appreciable in Fig. 3. Where well organized, adhered and uniform lens epithelial cells can be seen at the start of culture in A and B. While larger, elongated and unadhered mesenchymal cells can be observed on the posterior capsule in C and D after 36 days in culture, having migrated there from the anterior capsule.

It is interesting to note that even with our relatively high concentration of H_2_O_2_, in the cases where it did not prevent growth, but only significantly delayed it, the average speed at which samples reached total confluence, was similar to cultured controls. This shows the resilience and high proliferative capabilities of the surviving LECs. These results highlight the fine line between an effective and ineffective treatment and how difficult it is to find a 100% effective treatment.

Our results of 50% PCO prevention with H_2_Od treatment after one month were comparable or slightly better than earlier studies. One study^11^ with human tissue culture obtained 20% PCO prevention, while another *in vivo* study^13^ using rabbits, obtained 40% prevention. To our knowledge, the only study to obtain 100% PCO prevention with LEC death was with the use of 100 µM of Thapsigargin^11^, however this substance is highly cytotoxic.

The instances of cellular growth and PCO that were observed in treated samples, did appear significantly later than in cultured controls and after the end point (day 28) of other similar studies^11,12^. Based on this, future studies should aim to culture for longer than a month to assure results.

Histologically, there was a clear difference in the morphological aspect of cultured controls compared to treated samples (Fig. 7). These controls showed cells with well-defined nuclei and tended to form unorganized agglomerations of cells near the equator (Fig. 8A). In general, treated samples presented fewer cells which tended to be smaller and dispersed. In most H2O2 samples, only residual cell fragments with no visible nuclei were found. These fragments can still stain positively for both α-SMA and Vimentin (Fig. 8J,L). This was to be expected as both are found in the cytoskeleton of lens epithelial cells and Vimentin also in early lens fiber cells^32^.

It is known that both treatments severely damage cells, H2Od through hypotonic cell lysis^36^ and H2O2 through apoptosis or necrosis^24,37^. These processes are probably the origin of the residual cell fragments we see in our samples. Neither treatment seemed to prevent epithelial to mesenchymal transition since most samples expressed α-SMA, a known marker of this transition^29,30^. α-SMA also plays a role in capsular contraction during cell migration^29^. Its expression in our samples correlated with these functions, since it was present in all cases of wrinkling and intercapsular adhesion and staining was highest in areas of close intercapsular adhesion (Fig. 8B). Also, the only sample that did not present α-SMAs (C031) did not show capsular adhesion or wrinkling (Fig. 4).

In conclusion, hydrogen peroxide is a possible treatment for the delay of posterior capsule opacification, but the resilient nature of LECs makes this a difficult task.

## Electronic supplementary material

Supplementary Materials

## Data Availability

The data generated during and/or analyzed during the current study are available from the corresponding author on reasonable request.
