# Supplementary material for: Prevention of posterior capsule opacification through intracapsular hydrogen peroxide or distilled water treatment in human donor tissue
Source: Sci Rep. 2018 Aug 24;8:12739. doi: 10.1038/s41598-018-31178-y (PMC6109042; doi:10.1038/s41598-018-31178-y)
Supplement: Supplementary file 1 — Supplementary Materials [file 41598_2018_31178_MOESM1_ESM.pdf]

Prevention of posterior capsule opacification through intracapsular hydrogen peroxide or distilled water treatment in human donor tissue

Justin Christopher D’Antin[1,2], Rafael I. Barraquer[1,2,3], Francisco Tresserra[4], Ralph Michael[1,2]

1 Institut Universitari Barraquer, Universitat Autònoma de Barcelona, Barcelona, Spain

2 Centro de Oftalmología Barraquer, Barcelona, Spain

3 Universitat Internacional de Catalunya, Barcelona, Spain

4 Department of Pathology, Institut Universitari Dexeus, Barcelona, Spain

Corresponding author: Rafael I. Barraquer, Institut Universitari Barraquer, Laforja 88, E-08021 Barcelona, SPAIN; E-mail: prof.rafael@barraquer.com

Supplementary Table S1.

	Samples	Age (Years)	Sex	Postmortem Time (h)
**Uncultured Control**	**619B**	92	M	50
	**622B**	54	M	120
	**629A**	89	M	148

Table with the basic information of the three uncultured control samples.

Supplementary Figure S1.

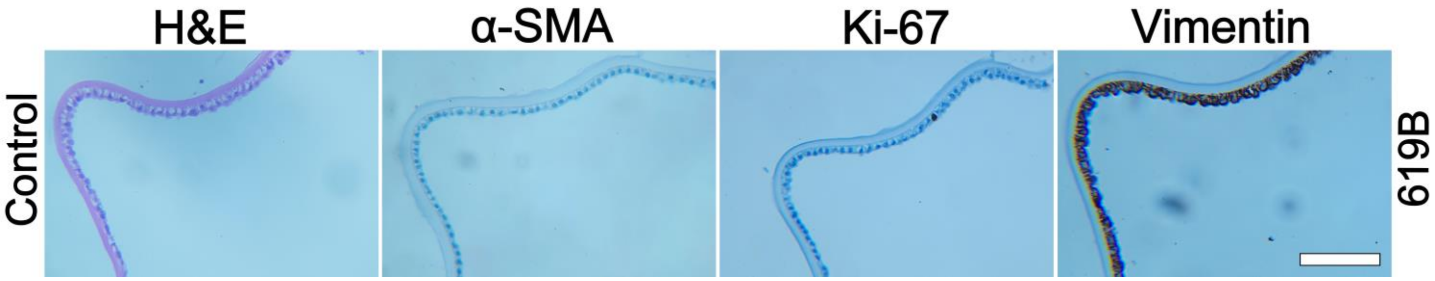

Microscopic images of the histological sections of one of our uncultured control sample 619B, showing staining for hematoxylin and eosin (H&E), alpha smooth muscle actin (α-SMA), ki-67 and Vimentin. Scale bars = 100 µm.

Supplementary Figure S2.

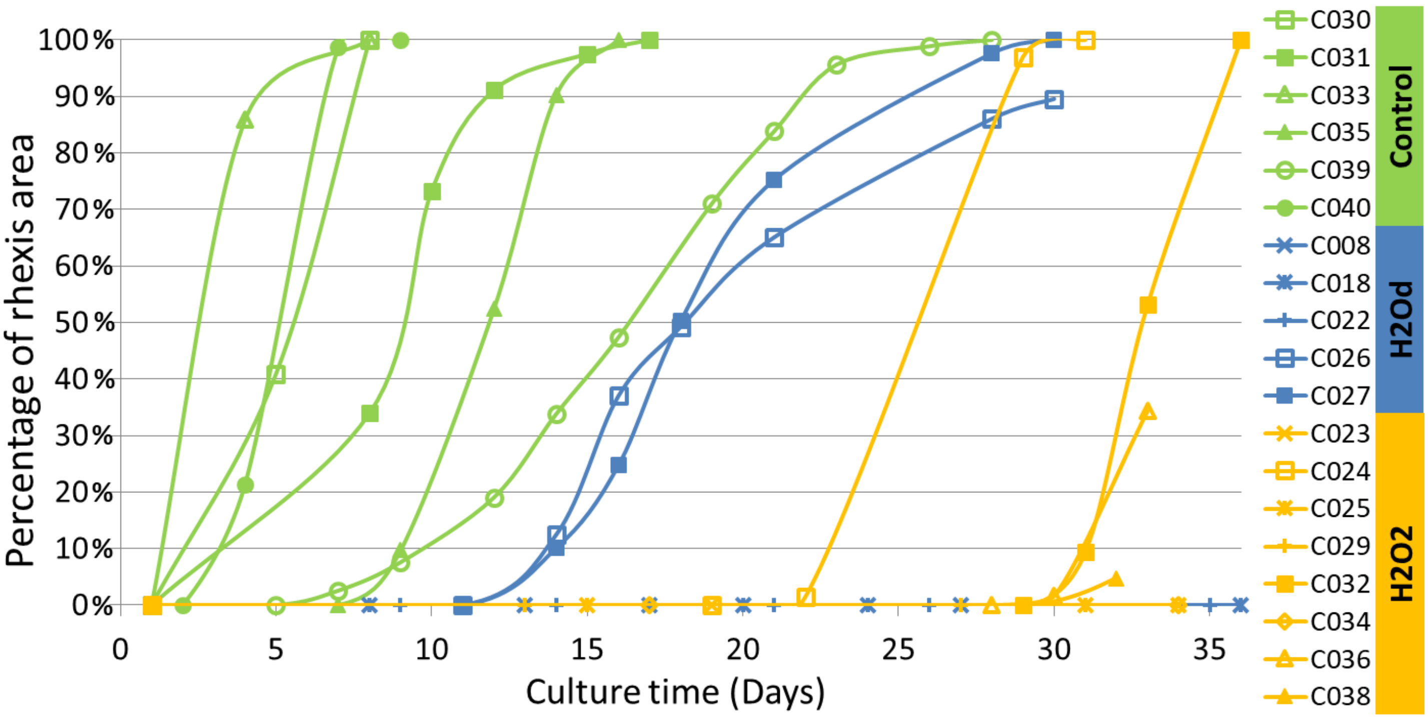

Diagram of cellular confluence progression during cell culture. Showing percentage of confluence on posterior capsule against culture time. 0% means no signs of cell growth on posterior capsule within visual limits of the rhexis and 100% means total confluence. Samples C008, C018, C022, C023, C025, C029 and C034 showed no signs of growth.

Supplementary Figure S3.

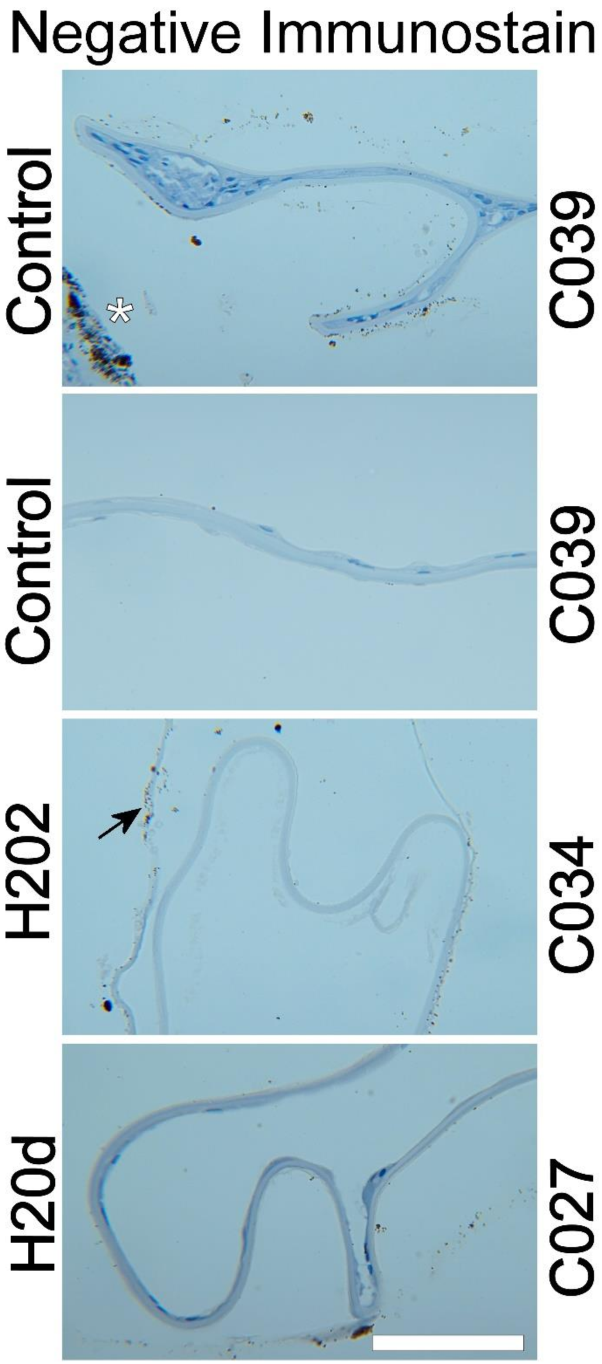

Histological sections of the negative immunostain controls of our samples. Immunostains processed with the BenchMark® ULTRA autostainer omitting the primary antibody. Within our capsule samples, no staining is observed. Asterisk show pigmented ciliary body next to control sample C039. Arrow points at zonule with adhered pigment Scale bars = 100µm.
